# Influence of time since injury and physical activity level on upper limb kinematics and muscle activation during wheelchair propulsion in complete T12/L1 spinal cord injury

**DOI:** 10.1186/s12891-025-08987-0

**Published:** 2025-08-21

**Authors:** San Hong, Hyunji Kim, Jooeun Ahn, Woojin Park

**Affiliations:** 1 Department of Industrial Engineering, Seoul National University, Seoul, South Korea; 2 Institute for Industrial Systems Innovation, Seoul National University, Seoul, South Korea; 3 Department of Physical Education, Seoul National University, Seoul, South Korea; 4 Soft Robotics Research Center, Seoul National University, Seoul, South Korea; 5 Institute of Sport Science, Seoul National University, Seoul, South Korea

**Keywords:** Biomechanics, Disability management, Electromyography, Environmental factors, Motion capture, Wheelchair propulsion

## Abstract

**Background:**

Wheelchair propulsion strategies vary widely among individuals with complete spinal cord injury (SCI), yet research typically focuses on differences between injury levels rather than variability within the same level. This study investigated how physical activity (PA) level and time since injury affect wheelchair propulsion biomechanics in individuals with identical T12/L1 SCI classification.

**Methods:**

Eleven participants with complete T12/L1 SCI performed wheelchair propulsion at maximum and self-selected speeds. Joint kinematics and electromyography data were collected from six upper body muscles. Participants were categorized by time since injury (Short: < 10 years, Medium: 10–25 years, Long: ≥ 25 years) and physical activity level (Low, Moderate, High). Group differences were analyzed using one-way ANOVA with Tukey post-hoc tests.

**Results:**

Significant differences in propulsion biomechanics were observed despite identical injury levels. Time since injury significantly affected elbow kinematics (*p* = 0.009) and biceps activity (*p* = 0.009), with the Long-time group showing increased elbow range of motion during maximum effort compared to the Medium-time group. PA level influenced trunk flexion (*p* = 0.036) and upper trapezius activity (*p* = 0.012), with the High-PA group demonstrating greater trunk flexion during maximum effort compared to the Low-PA group. Notably, propulsion velocity showed no significant differences between groups.

**Conclusions:**

Wheelchair propulsion strategies vary significantly among individuals with identical SCI level, demonstrating that neurological classification alone does not determine movement patterns. The adaptive strategies observed in experienced and physically active wheelchair users suggest rehabilitation approaches should consider these factors beyond injury classification. These findings highlight the importance of promoting long-term PA participation and ongoing biomechanical adaptation throughout the lifespan of individuals with SCI.

## Background

Wheelchair propulsion strategies among individuals with spinal cord injuries (SCI) represent a critical research focus in rehabilitation, as inefficient techniques can lead to upper extremity overuse injuries, decreased mobility, and reduced community participation. Understanding optimal strategies is essential for developing effective rehabilitation programs that promote long-term health and functional independence in this population.

Research has traditionally focused on comparing wheelchair propulsion strategies across different SCI classification levels, with studies consistently demonstrating that injury level significantly influences feasible movement patterns. These investigations typically categorize the SCI population into broad groups (non-disabled, paraplegic, tetraplegic) and analyze joint angles and movement patterns to characterize propulsion kinematics [6, 16, 19, 22, 23, 27, 29, 37]. This approach aligns with conventional clinical practice that primarily uses neurological classification to predict functional capabilities and guide rehabilitation interventions.

However, a significant gap exists in understanding the considerable variability observed within the same injury level. Clinical experience suggests that individuals with identical SCI classifications often exhibit markedly different propulsion strategies and functional outcomes, which may be attributed to personal factors like time since injury and physical activity (PA) level. While research has established that PA and sports experience influence postural strategies in various populations [9, 11, 31, 42], and that people with SCI develop unique movement patterns to enhance functional performance [2], the relationship between these personal variables and wheelchair propulsion biomechanics remains largely unexplored, particularly among individuals with similar impairment levels.

Understanding how time since injury and PA level influence propulsion biomechanics offers valuable insights for developing evidence-based, personalized training interventions. This knowledge challenges the prevalent view of SCI as a static condition with predetermined functional limitations, instead supporting a dynamic, lifespan approach to disability management that recognizes continued adaptation potential. By identifying specific kinematic and muscle activation patterns in physically active wheelchair users with extended time since injury, rehabilitation professionals can design targeted programs that optimize propulsion efficiency, potentially reduce injury risk, and accelerate PA participation among less active individuals. These insights could guide the development of comprehensive management approaches that address the evolving nature of movement adaptations throughout the lifespan, with implications for healthcare resource allocation and policy development. Ultimately, this approach may enhance physical health, support greater community participation, and improve quality of life for individuals with SCI beyond the acute rehabilitation phase.

Therefore, this study aimed to investigate the relationship between wheelchair propulsion strategies and two personal variables—time since injury and PA level—by examining a sample of individuals with identical T12/L1 SCI classification. Propulsion strategies were quantitatively characterized using joint kinematics and electromyography (EMG) measures to provide objective evidence for how these personal factors influence movement patterns independent of neurological impairment level.

## Methods

### Participants

Eleven participants (7 males, 4 females; age range: 25–63 years) with T12/L1 SCI classified under American Spinal Injury Association (ASIA) Impairment Scale A score (complete injury) were recruited. Participants were recruited through outreach via national networks affiliated with the Korea Spinal Cord Injury Association. To minimize the potential influence of pain on propulsion biomechanics, only individuals who reported no shoulder pain during wheelchair propulsion were eligible for participation. Participants’ anthropometric data were collected prior to the experiment, with seated heights varying between 70 and 90 cm and weights ranging from 40 to 80 kg. PA levels were determined using the International Physical Activity Questionnaire (IPAQ) [3], where participants reported PA at work, in daily life, and in leisure and professional sports. The IPAQ is a widely used self-report instrument designed to assess PA across multiple domains and to categorize individuals into low, moderate, or high activity levels. Although originally developed for the general population, recent studies has supported its use and comparability in populations with SCI [10, 18]. Based on their responses, participants were categorized into three PA levels: Low (inactive or minimal PA required in daily life, equivalent to < 600 MET-minutes/week; *n* = 4), Moderate (regular engagement in recreational PA or sports, 600–3000 MET-minutes/week; *n* = 3), and High (high-intensity PA required at work or engagement in professional sports, > 3000 MET-minutes/week; *n* = 4). Additionally, participants were categorized based on the time since injury: Short (< 10 years; *n* = 3), Medium (10–25 years; *n* = 4), and Long (≥ 25 years; *n* = 4). Although no universally accepted criteria exist for categorizing time since injury in SCI populations, previous studies have adopted time-based classifications guided by clinical reasoning or research objectives [14, 21, 39]. In this study, participants were categorized into three groups: < 10 years, 10–25 years, and ≥ 25 years post-injury. These groupings were selected to align with the study’s objective of examining long-term patterns in wheelchair propulsion strategies within the context of SCI rehabilitation in South Korea. Unlike Western countries where community reintegration typically occurs within several months, individuals with SCI in South Korea often experience prolonged hospitalization—averaging approximately 30 months, and in some cases extending up to 10 years [12, 13, 30]. During this extended period, patients receive little to no wheelchair training. As a result, most individuals begin learning to use a wheelchair only after discharge, often through informal peer support. Accordingly, the < 10-year category in this study represents an early post-discharge phase of wheelchair use and skill development, distinguishing it meaningfully from the longer-term experience captured by the other groups.

### Data collection

Participants in this study propelled their wheelchairs using a wheelchair roller ergometer (Wheely-X, Kangsters Inc., Republic of Korea) and participated in two tasks: 1) 15 s of forward propulsion at their maximum speed (MAX), and 2) 30 s of forward propulsion at a self-selected speed (SEL). The MAX task was designed to simulate not just sprinting but also challenging conditions like uphill gradients and rough road surfaces. This approach aimed to observe the propulsion strategies employed by participants under various situational demands. Participants had ample time to acclimatize on the roller ergometer until they indicated they were ready to perform the task. They performed each task three times. The order of the task trials was randomized for each participant. If any participant reported pain or discomfort during the trial, the task was immediately terminated to prevent risk of aggravation.

Participants performed the tasks using their personal wheelchairs, all of which were manual and equipped with 24-inch wheels. To maintain consistency in the experimental setup and minimize the potential impact of wheelchair size variability on the collected data, each wheelchair was adjusted to ensure that the participants'hands were aligned with the center of the wheel when seated upright.

**Fig. 1:**
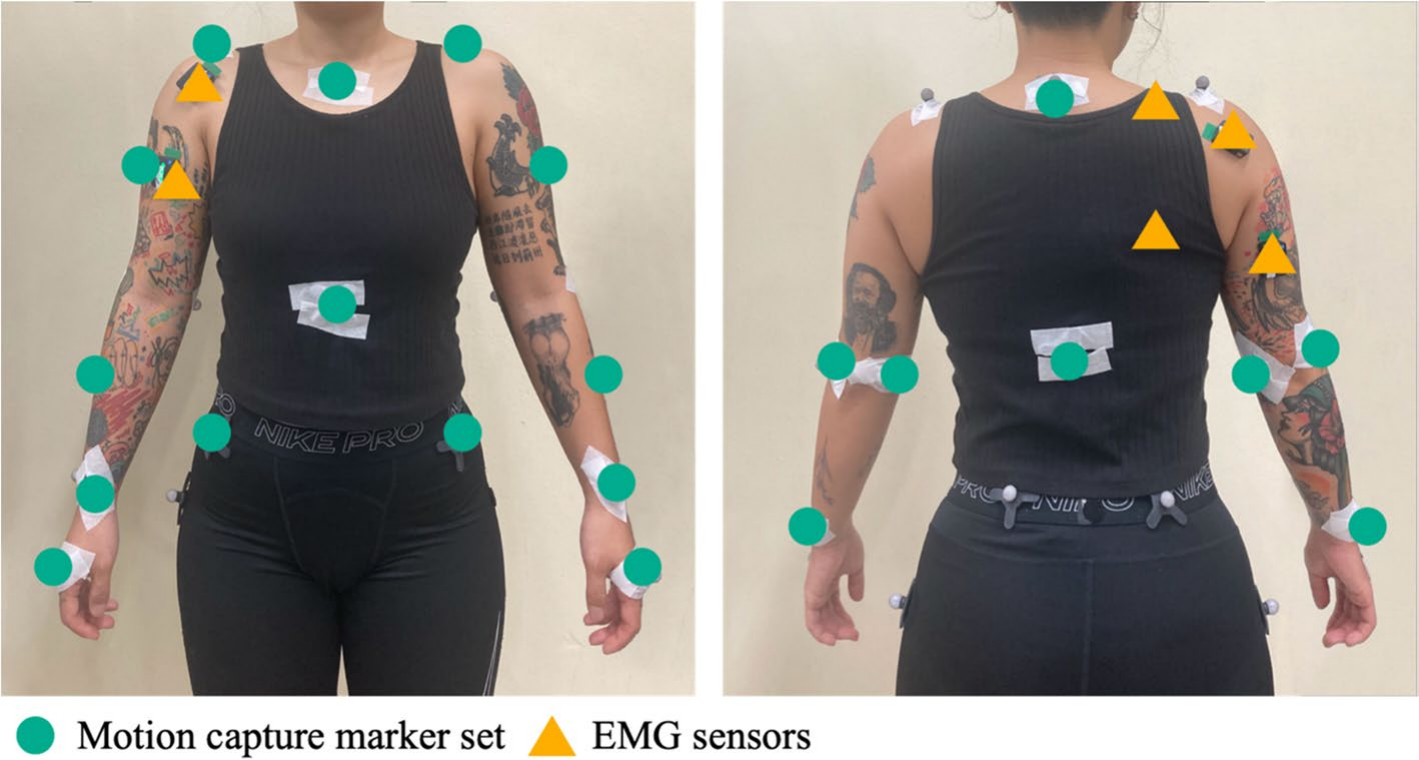
Placement of marker set and EMG Sensors

Motion capture system (Optitrack Prime13, NaturalPoint Inc., USA) was used to collect kinematic data of upper limb joint and trunk angles. Twenty-two passive retroreflective markers (18 plug in gait marker set with four tracking markers) were placed on anatomical landmarks on the torso and upper extremities [28, 40], and wireless EMG system (Trigno TM, Delsys Inc., USA) was used to collect unilateral activity of six muscles: biceps brachii (Biceps), triceps brachii (Triceps), anterior deltoid (Ant.Delt.), posterior deltoid (Post.Delt.), upper trapezius (Up.Trap.), and lower trapezius (Low.Trap) (Fig. 1). These muscles were selected based on their primary roles in wheelchair propulsion: the Biceps and Triceps control elbow flexion/extension, the Ant.Delt. and Post.Delt. contribute to shoulder movement during the push and recovery phases, and the Up.Trap. and Low.Trap. muscles are involved in scapular stability and trunk control during propulsion. Additionally, the whole experimental process was video-recorded from sagittal and frontal plane with iPhone 13 for propulsion velocity analysis.

### Data analysis

**Fig. 2:**
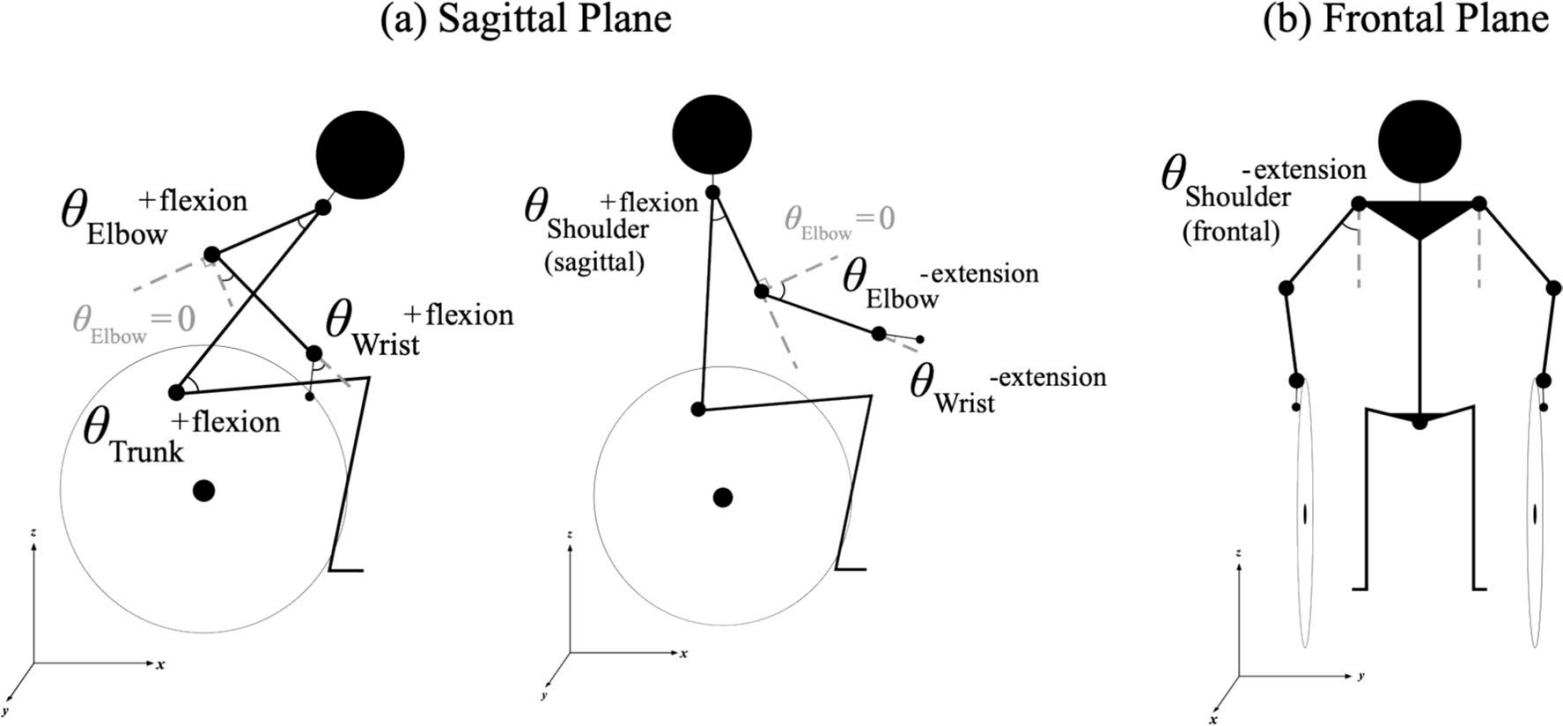
Angle definitions for the propulsion model

Marker trajectories were processed in Optitrack software and modeled with Visual3D (C-motion, USA) software to acquire six upper limb joint and trunk angles: shoulder (referred as sho_sag in Fig. 2), elbow, wrist, and trunk angles in the sagittal plane (Fig. 2(a)); shoulder (referred as sho_fro in Fig. 2) in the frontal plane (Fig. 2(b)).

The cycles were segmented at the point of hand contact with the wheel and then normalized on a scale from 0 to 100%. From the total cycles recorded, those associated with starting and stopping strokes were excluded to negate the effects of acceleration, and the central five cycles were selected for detailed analysis. The maximum and minimum peak angles for the shoulder, elbow, wrist joints, and trunk were identified. Subsequently, the observed range of joint angle (ORJA) was determined by calculating the difference between these maximum and minimum angles. We analyzed the propulsion strategy for each task with five indicators from the peak joint angle analysis. These indicators were selected to capture both the absolute kinematic characteristics during each task and the relative changes in movement patterns between maximum and self-selected speeds, which can suggest adaptive strategies employed by wheelchair users. The five indicators are as follows:

- The ORJA during a wheelchair propulsion cycle in the MAX task (ORJA_MAX),
- The ORJA in the SEL task (ORJA_SEL),
- The difference between ORJA_MAX and ORJA_SEL (MAX-SEL ORJA),
- The difference between the maximum joint angle positions for MAX and SEL (MAX-SEL Peak), and
- The difference between the minimum joint angle positions for MAX and SEL (MAX-SEL Valley).

The EMG data was subjected to a bandpass filter, incorporating low-pass filtering at 450 Hz and high-pass filtering at 20 Hz, to minimize noise interference. For normalization within a dynamic movement cycle (DMC), sinusoidal rectification was implemented over a 0.01-s interval to accurately identify the maximum EMG value. The normalization process was then carried out using the following formula:

$${\%DMC }= {\text{EMG}}_{\text{Cycle}} / {\text{EMG}}_{\text{DMC}}$$

where EMG_Cycle_ represents the average of maximum EMG value of five cycles each, and EMG_DMC_ denotes the maximum EMG value recorded during the propulsion phase. We analyzed the propulsion strategy for each task with three indicators. These EMG indicators were selected to quantify muscle activation patterns during different propulsion intensities and to examine how participants modulate their muscle recruitment strategies when transitioning between self-selected and maximum speed conditions. The three indicators are as follows:

- The average of the peak %DMC EMG amplitudes during a wheelchair propulsion cycle in the MAX task (%DMC_MAX),
- The average of the peak %DMC EMG amplitudes in the SEL task (%DMC_SEL), and
- The difference between %DMC_MAX and %DMC_SEL, (MAX-SEL %DMC).

The collected data met the conditions of normality and homogeneity of variances, thus a One-Way analysis of variance (ANOVA) followed by a Tukey post hoc test was performed to determine the effect of each of the time since injury and PA level factors on the propulsion strategy indicators.

In addition, to measure the propulsion velocity of the five extracted cycles, we calculated the revolutions per minute (RPM) of a 24-inch wheel from the recorded video. The collected data met the conditions of normality and homogeneity of variances, thus a One-Way analysis of variance (ANOVA) followed by a Tukey post hoc test was performed to determine the effect of each of the time since injury and PA level factors on the propulsion strategy indicators.

In addition, to measure the propulsion velocity of the five extracted cycles, we calculated the RPM of a 24-inch wheel from the recorded video.

## Results

### Range of motion (ORJA)

Time since injury had a significant relationship with elbow kinematics during wheelchair propulsion. The difference in elbow range of motion between maximum speed and self-selected speed (MAX-SEL ORJA) showed significant variation across time since injury groups (*p* = 0.009). A Tukey post hoc test showed significant differences between Medium- and Long-time groups (mean difference = −33.2°, *p* = 0.008). The Medium-time group exhibited a smaller range of motion during maximum speed propulsion compared to their self-selected pace (mean = −24.4° ± 10.2°), while the Long-time group showed increased range of motion under maximum effort (mean = 8.77° ± 8.66°) (See Tables 1 and 2).

**Table 1:** One-way ANOVA and descriptive statistics of joint angles’ indicators (right upper extremity)

Group	Indicator	*Shoulder (Sagittal)*	*Shoulder (Frontal)*	*Elbow*	*Wrist*	*Trunk*
Time Since Injury	ORJA_MAX	0.41	0.346	0.112	0.342	0.648
	ORJA_SEL	0.434	0.69	0.545	0.321	0.286
	MAX-SEL ORJA	0.719	0.05	0.009^**^	0.641	0.829
	MAX-SEL Peak	0.816	0.174	0.074	0.37	0.618
	MAX-SEL Valley	0.537	0.744	0.757	0.333	0.696
Indicator			Group	Short	Medium	Long
MAX-SEL ORJA			Time Since Injury	Mean(SD)	Mean(SD)	Mean(SD)
			*Elbow*	−5.44° (15.2)	−24.4° (10.2)	8.77° (8.66)
Group	Indicator	*Shoulder (Sagittal)*	*Shoulder (Frontal)*	*Elbow*	*Wrist*	*Trunk*
PA Level	ORJA_MAX	0.876	0.639	0.444	0.803	0.937
	ORJA_SEL	0.433	0.669	0.836	0.254	0.524
	MAX-SEL	0.679	0.937	0.053	0.628	0.902
	ORJA					
	MAX-SEL Peak	0.649	0.297	0.043	0.544	0.057
	MAX-SEL Valley	0.763	0.462	0.693	0.482	0.036^*^
Indicator			Group	Low	Moderate	High
MAX-SEL Valley			PA Level	Mean(SD)	Mean(SD)	Mean(SD)
			*Trunk*	−2.94° (0.357)	−17.8° (16.8)	−23.7° (6.61)

^*^p < 0.05

^**^p < 0.01

**Table 2:** Tukey post-hoc test of Table 1 (Right upper extremity)

Group: Time Since Injury				
MAX-SEL ORJA	Elbow			
		*Short*	*Medium*	*Long*
*Short*	Mean difference	—	18.9°	−14.2°
	*p*-value	—	0.127°	0.276°
*Medium*	Mean difference		—	−33.2°^**^
	*p*-value		—	0.008
*Long*	Mean difference			—
	*p*-value			—
Group: PA Level				
MAX-SEL Valley	Trunk			
		*Low*	*Moderate*	*High*
*Low*	Mean difference	—	−14.8°	−20.75°^*^
	*p*-value	—	0.155	0.033
*Moderate*	Mean difference		—	−5.90°
	*p*-value		—	0.697
*High*	Mean difference			—
	*p*-value			—

^*^p < 0.05

^**^p < 0.01

PA level was significantly associated with trunk kinematics. Specifically, the MAX-SEL Valley of trunk angle differed between Low- and High-PA level groups (p = 0.036). The High-PA group exhibited larger trunk flexion under maximum effort (mean = −23.7° ± 6.61°) compared to the Low-PA group (mean = −2.94° ± 0.357°) (See Tables 1 and 2).

### Muscle activity

One-way ANOVA demonstrated significant associations between time since injury and biceps muscle activity. Biceps activity differed significantly in both the MAX task (*p* = 0.009) and in the difference between MAX and SEL tasks (MAX-SEL %DMC, *p* = 0.034). Post-hoc analysis indicated significant differences between Short- and Long-time groups (mean difference = −0.223, *p* = 0.007 for %DMC_MAX; mean difference = −0.309, *p* = 0.029 for MAX-SEL %DMC). The Long-time group showed higher biceps activity during maximum propulsion (mean = 0.734 ± 0.037) compared to the Short group (mean = 0.511 ± 0.096), as well as greater change between task conditions (0.478 ± 0.094 vs. 0.169 ± 0.114) (See Tables 3 and 4).

**Table 3:** One-way ANOVA and descriptive statistics EMG indicators (Right upper extremity)

Group	Indicator	*Biceps*	*Triceps*	*Ant. Delt*	*Post. Delt*	*Up. Trap*	*Low. Trap*
Time Since Injury	%DMC_MAX	0.009^*^	0.529	0.834	0.41	0.344	0.744
	%DMC_SEL	0.782	0.684	0.135	0.549	0.146	0.471
	MAX-SEL %DMC	0.034^*^	0.939	0.057	0.121	0.05	0.486
Indicator				Group	Short	Medium	Long
				Time Since Injury	Mean(SD)	Mean(SD)	Mean(SD)
%DMC_MAX				*Biceps*	0.511(0.096)	0.616(0.072)	0.734(0.037)
MAX-SEL %DMC				*Biceps*	0.169(0.114)	0.31(0.156)	0.478(0.094)
Group	Indicator	*Biceps*	*Triceps*	*Ant. Delt*	*Post. Delt*	*Up. Trap*	*Low. Trap*
PA Level	%DMC_MAX	0.083	0.595	0.223	0.975	0.012^*^	0.511
	%DMC_SEL	0.573	0.958	0.506	0.235	0.443	0.417
	MAX-SEL%DMC	0.735	0.725	0.044^*^	0.393	0.051	0.208
Indicator				Group	Low	Moderate	High
				PA Level	Mean(SD)	Mean(SD)	Mean(SD)
%DMC_MAX				*Up.Trap*	0.31(0.148)	0.634(0.183)	0.224(0.080)
MAX-SEL %DMC				*Ant. Delt*	0.147(0.231)	0.534(0.097)	0.178(0.161)

^*^p < 0.05

^**^p < 0.01

**Table 4:** Tukey post-hoc test of Table 3 (Right upper extremity)

Group: Time Since Injury					
%DMC_MAX	Biceps				
		*Short*		*Medium*	*Long*
*Short*	Mean difference	—		−0.105	−0.223^**^
	*p*-value	—		0.18	0.007
*Medium*	Mean difference			—	−0.118
	*p*-value			—	0.096
*Long*	Mean difference				—
	*p*-value				—
MAX-SEL %DMC	Biceps				
			*Short*	*Medium*	*Long*
*Short*	Mean difference		—	−0.142	−0.309^*^
	*p*-value		—	0.349	0.029
*Medium*	Mean difference			—	−0.167
	*p*-value			—	0.205
*Long*	Mean difference				—
	*p*-value				—
Group: PA Level					
%DMC_MAX	Up.Trap				
			*Low*	*Moderate*	*High*
*Low*	Mean difference		—	−0.324^*^	0.0859
	*p*-value		—	0.036	0.666
*Moderate*	Mean difference			—	0.4096^*^
	*p*-value			—	0.011
*High*	Mean difference				—
	*p*-value				—
MAX-SEL %DMC	Ant.Delt				
			*Low*	*Moderate*	*High*
*Low*	Mean difference		—	−0.388	−0.0313
	p-value		—	0.052	0.967
*Moderate*	Mean difference			—	0.3564
	*p*-value			—	0.072
*High*	Mean difference				—
	*p*-value				—

^*^p < 0.05

^**^p < 0.01

PA level also influenced muscle activity. Upper trapezius (Up.Trap.) activation during the MAX task showed significant variation across PA levels (*p* = 0.012), with post-hoc differences observed between the Low- and Moderate-PA groups (mean difference = −0.324, *p* = 0.036) and between Moderate- and High-PA groups (mean difference = 0.4096, *p* = 0.011). The Moderate-PA group exhibited the highest Up.Trap. activation (mean = 0.634 ± 0.183), compared to Low (mean = 0.31 ± 0.148) and High PA groups (mean = 0.224 ± 0.080). Although ANOVA revealed a significant effect of PA level on anterior deltoid (Ant.Delt.) activity in MAX-SEL %DMC (*p* = 0.044), no post-hoc comparisons reached statistical significance. Descriptively, the Moderate-PA group showed higher values (mean = 0.534 ± 0.097) compared to Low-PA (mean = 0.147 ± 0.231) and High-PA groups (mean = 0.178 ± 0.161) (See Table 3 and 4).

### Propulsion velocity

No statistically significant differences in propulsion velocity (RPM) were found between groups categorized by either time since injury or PA level.

**Fig. 3:**
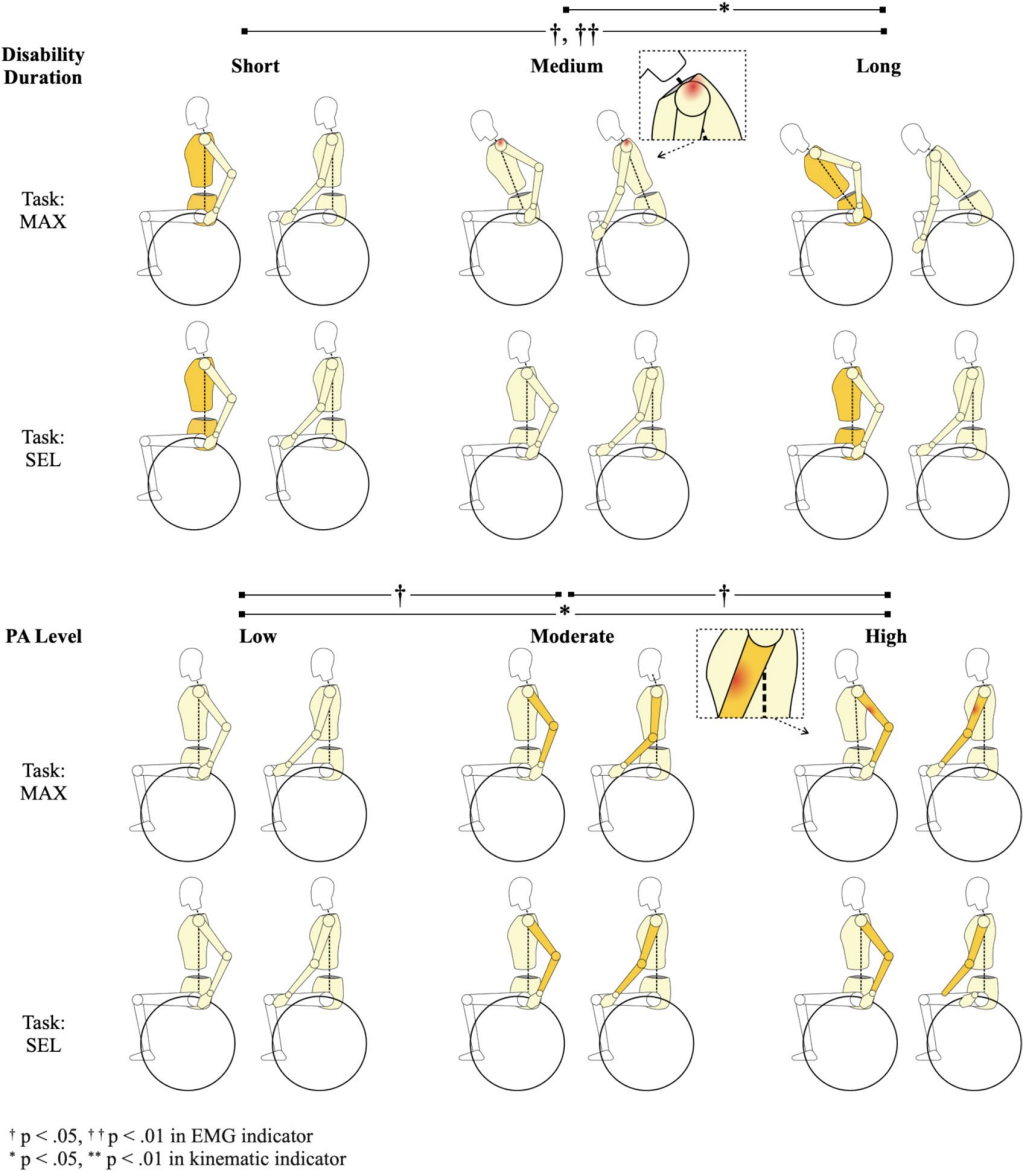
Comparison of wheelchair propulsion kinematics and muscle activity across time since injury and PA level groups. Note. Darker yellow highlights differences in joint kinematic variables. Red highlights differences in muscle activity

Figure 3 presents the differences in kinematic and EMG measures across time since injury and PA level groups. Joint angle differences are color-coded in yellow, while EMG differences are marked in red. The upper panel visualizes elbow ORJA and biceps %DMC differences across time since injury groups. The lower panel highlights trunk angle differences and upper trapezius activity across PA levels.

## Discussion

This study provides a pioneering investigation of propulsion biomechanics variability among individuals with complete T12/L1 SCI. By controlling for injury level, we observed substantial variation in wheelchair propulsion kinematics and muscle activity even within a specific neurological impairment category. Our findings revealed distinct kinematic and muscle activation patterns associated with time since injury and PA level, suggesting that multiple factors beyond neurological classification contribute to movement strategies in wheelchair users.

Time since injury showed a significant relationship with elbow kinematics and biceps muscle activity, with the Long-time group demonstrating different movement patterns compared to those with shorter time since injury. These differences suggest that individuals adapt their elbow movement strategies differently as they accumulate experience with wheelchair use. Despite these distinct kinematic differences, propulsion velocity showed no significant differences between groups, suggesting participants achieve similar performance outcomes through different strategies in kinematics and muscle activity. This contrast suggests a fundamental difference in propulsion strategy adaptation; experienced users appear to develop techniques that leverage greater elbow mobility when performing high-intensity propulsion. This finding indicates that longer time since injury is associated with greater disparity in biceps muscle activation between MAX and SEL tasks. It is plausible that enhanced neuromuscular control has been developed through years of wheelchair propulsion. This increased biceps engagement in the Long-time group may represent a neuromuscular adaptation developed over years of wheelchair use, allowing for more effective force generation during propulsion.

PA level was associated with distinct trunk kinematics and upper trapezius muscle activation patterns, with the High-PA group exhibiting greater trunk flexion during maximum effort propulsion compared to the Low-PA group. While Newsam et al. [20] reported relatively consistent trunk motion across different SCI levels, our results demonstrate considerable variability in trunk positioning strategies among individuals with identical injury levels but different PA profiles. This variability aligns with Rodgers et al.'s identification of trunk flexion as an important adaptive movement during propulsion under fatigue [25]. Our findings extend this concept, revealing that High-PA individuals with complete T12/L1 SCI demonstrate significantly greater trunk flexion during maximum effort. This strategic use of trunk movement suggests that physically active individuals have developed more sophisticated biomechanical techniques to maximize propulsion efficiency under challenging conditions. This finding suggests that individuals with higher PA levels utilize trunk flexion more strategically during high-intensity propulsion, likely representing a learned technique to generate additional power when needed.

We also observed a distinct pattern of shoulder muscle activation. The distinct pattern of higher Up.Trap. activation in the Moderate-PA group compared to both Low-PA and High-PA groups suggests this population may rely more heavily on shoulder girdle musculature during intense propulsion efforts, while the High-PA group potentially distributes effort more effectively across multiple muscle groups. Furthermore, the Moderate group exhibits enhanced Up.Trap. activation during MAX task execution compared to both Low and High PA groups, potentially indicating a transitional propulsion strategy that relies more heavily on shoulder stabilization before developing the more advanced techniques observed in the high-activity group.

These observations challenge and complement the traditional paradigm in wheelchair biomechanics research that primarily categorizes movement patterns based on injury level alone. Our findings add to Leving et al.'s observations that mechanical efficiency differed significantly between users with varying experience levels, while kinetic variables of propulsion did not show clear differences [15]. Similarly, Vanlandewijck et al. [38] demonstrated that force application patterns in wheelchair propulsion represent adaptive strategies that individuals develop over time rather than direct consequences of neurological impairment. Both previous research and our findings provide compelling evidence that wheelchair propulsion patterns are determined largely by post-injury adaptations rather than being solely dictated by neurological impairment. The consistent propulsion velocity observed across groups despite varied movement strategies suggests that wheelchair users develop individualized propulsion techniques that achieve similar functional outcomes through different biomechanical pathways, effectively compensating for their specific constraints.

These insights have important implications for rehabilitation approaches. Traditional models have emphasized injury level as the primary determinant of function, overlooking significant adaptations that occur beyond neurological classification. Our findings suggest comprehensive assessment should consider both time since injury and PA history to fully understand movement capabilities. Importantly, the biomechanical differences observed between PA level groups suggest that even individuals with long-term disabilities may benefit from targeted PA interventions to develop enhanced movement patterns, as demonstrated by the kinematic and muscle activity differences observed among participants with identical injury levels but varying PA levels. These observed differences challenge the conventional view of SCI as a static condition with limited potential for functional advancement beyond initial recovery.

Our findings provide practical insights for wheelchair propulsion training and lifelong disability management. The significant adaptations observed across different groups have implications that extend well beyond initial rehabilitation. The High-PA group demonstrated remarkable trunk adaptation during maximum effort conditions (−23.7° ± 6.61° trunk flexion compared to −2.94° ± 0.357° in the Low-PA group), suggesting superior situational adaptation when facing fatigue-inducing challenges. Similarly, the Long-time group exhibited expertise in optimizing handrim contact and release positions, as evidenced by their elbow range of motion patterns. These naturally developed strategies represent critical knowledge that can inform targeted training interventions for individuals with lower PA levels or shorter time since injury, focusing specifically on trunk engagement and situation-specific propulsion techniques. These biomechanical adaptations may contribute to improved functional outcomes after discharge, potentially supporting greater independence in community environments and comprehensive self-management during daily activities [24, 36]

These training approaches are particularly relevant given that current healthcare systems in South Korea typically allocate minimal resources for long-term community reintegration after discharge, focusing instead on acute care [33, 41]. Our results provide evidence-based justification for extending rehabilitation support beyond the acute phase, with particular emphasis on promoting sustained PA participation. The differential movement patterns observed between PA groups suggests that policy frameworks should recognize post-discharge PA programs as essential components of comprehensive SCI management that facilitate continued functional improvement throughout the lifespan [1, 7, 8, 35].

Beyond PA and time since injury, other individual characteristics likely contribute to the diverse movement strategies observed, including anthropometrics, pre-injury motor preferences, psychological factors, and unique anatomical characteristics. While previous research has categorized wheelchair users primarily according to neurological impairment kinematics [6, 16, 19, 22, 23, 27, 29, 37], our study conclusively demonstrates that factors beyond neural damage—particularly PA level and time since injury—play crucial roles in determining movement patterns. Rehabilitation programs should therefore acknowledge diverse strategies within the same SCI level and provide individualized approaches that recognize the ongoing potential for positive adaptations through appropriate PA interventions, regardless of how long ago the disability occurred [17, 26, 32, 34].

Several limitations and questions remain for future investigation. Our cross-sectional design cannot establish causal relationships or track adaptation development over time. Longitudinal studies following individuals from acute injury through long-term adaptation would provide valuable insights into how propulsion strategies evolve and which environmental factors most significantly influence this development. Additionally, our study focused on kinematic and muscle activation patterns without examining metabolic efficiency, joint loading, or pain outcomes. Furthermore, we did not assess other potentially influential personal variables such as detailed anthropometric characteristics, pain levels, psychological factors, or wheelchair-specific configurations, which may contribute significantly to the movement strategy variations observed and warrant investigation in future studies. We also acknowledge that differences in wheelchair configuration (e.g., seat slope, backrest height, or custom adjustments) were not analyzed in this study. Although all participants used their personal wheelchairs to preserve ecological validity, these unmeasured variables may have contributed to variations in propulsion strategy. Future studies should account for such equipment-related factors to better isolate the impact of personal and behavioral variables.

 In addition to biomechanical insights, future research should incorporate clinical outcome measures to better evaluate the functional implications of propulsion strategies. Tools such as the Wheelchair User’s Shoulder Pain Index [4, 5] could clarify whether observed kinematic and muscle activation differences are associated with clinically meaningful outcomes such as pain reduction, propulsion efficiency, or independence in daily life. Incorporating both biomechanical and functional data would further strengthen the translational value of future studies and inform the development of targeted interventions with measurable clinical benefits. Another limitation is our sample size, which necessitates caution when generalizing these findings. Despite this, the statistically significant differences observed provide compelling initial evidence for the importance of considering these factors. Future research should investigate whether the different propulsion strategies observed within the same injury level have implications for long-term joint health and functional performance.

## Conclusions

This study demonstrates that wheelchair propulsion strategies among individuals with identical T12/L1 SCI classification vary significantly based on time since injury and PA level. Our findings challenge the traditional rehabilitation paradigm that primarily uses neurological classification to predict functional capabilities by showing that propulsion biomechanics are influenced substantially by factors beyond injury level. The distinct movement patterns observed in long-time and highly physically active participants reflect continued adaptation potential throughout the lifespan, with experienced wheelchair users developing more efficient elbow range of motion strategies and physically active users demonstrating strategic trunk flexion during challenging conditions. These naturally developed adaptations represent valuable knowledge that could inform evidence-based, targeted interventions for individuals with SCI. By recognizing these differences and incorporating them into rehabilitation approaches, healthcare providers can better support continued functional improvement beyond acute care, potentially enhancing independence, community participation, and quality of life for individuals with SCI. Future rehabilitation policy and practice should consider time since injury and PA history as essential components of comprehensive assessment and intervention planning, moving beyond a static view of SCI toward a dynamic, lifespan approach to disability management.

## Data Availability

The dataset used and analyzed during the current study is available from the corresponding author on reasonable request.

## Abbreviations

SCI: Spinal cord injury
PA: Physical activity
EMG: Electromyography
ORJA: Observed range of joint angle
MAX: Maximum speed propulsion task
SEL: Self-selected speed propulsion task
MAX-SEL: Difference between maximum and self-selected speed measurements
MAX-SEL ORJA: Difference between observed range of joint angle during maximum and self-selected speed
MAX-SEL Peak: Difference between maximum joint angle positions for maximum and self-selected speed
MAX-SEL Valley: Difference between minimum joint angle positions for maximum and self-selected speed
%DMC: Percentage of dynamic movement cycle
%DMC_MAX: Average of peak normalized EMG amplitudes during maximum speed task
%DMC_SEL: Average of peak normalized EMG amplitudes during self-selected speed task
Ant.Delt.: Anterior deltoid
Post.Delt.: Posterior deltoid
Up.Trap.: Upper trapezius
Low.Trap.: Lower trapezius
ANOVA: Analysis of variance
RPM: Revolutions per minute
MAX-SEL %DMC: Difference between normalized EMG amplitudes in maximum and self-selected speed
